# Endoscopic Immuno-Oncology: A New Frontier in Treatment of Pancreatic Cancer

**DOI:** 10.3390/cancers17132091

**Published:** 2025-06-23

**Authors:** Varun Vemulapalli, Cristina Natha, Nirav Thosani

**Affiliations:** 1 Department of Internal Medicine, The University of Texas Health Science Center at Houston, Houston, TX 77030, USA; varun.vemulapalli@uth.tmc.edu (V.V.); cristina.m.natha@uth.tmc.edu (C.N.); 2 Department of Surgery and Interventional Gastroenterology, The University of Texas Health Science Center and McGovern School of Medicine, Houston, TX 77030, USA

**Keywords:** pancreatic ductal adenocarcinoma (PDAC), radiofrequency ablation (RFA), tumor microenvironment (TME)

## Abstract

Pancreatic ductal adenocarcinoma (PDAC) remains one of the most challenging malignancies to treat, largely due to late diagnosis, limited surgical options, and profound resistance to systemic therapies, all of which contribute to a five-year survival rate of approximately 9%. The dense, hypoxic, and immunosuppressive tumor microenvironment (TME) of PDAC plays a critical role in immune evasion and therapeutic failure. As conventional immunotherapies have shown limited efficacy in PDAC, attention has shifted toward local interventions capable of remodeling the TME to enhance immune responsiveness. Endoscopic ultrasound (EUS)-guided ablative therapies have emerged as promising strategies to overcome these barriers. A range of modalities—including irreversible electroporation, microwave ablation, photodynamic therapy, and cryoablation—are under investigation. Among these, radiofrequency ablation (RFA) stands out as the most promising therapy as it not only induces direct tumor cytoreduction but also promotes antigen release, triggers immunogenic cell death, and may work synergistically with systemic therapies such as immune checkpoint inhibitors and adenosine pathway blockade. This review aims to explore the evolving role of endoscopic ablative therapies with a focus on RFA as a potential platform for immune activation in pancreatic cancer.

## Simple Summary

Pancreatic ductal adenocarcinoma (PDAC) is a highly aggressive malignancy due to late-stage diagnosis, limited surgical eligibility, and resistance to systemic therapies. The tumor microenvironment (TME) in PDAC is uniquely dense and immunosuppressive, limiting effective immune infiltration and therapeutic response. This review discusses the emerging role of endoscopic ultrasound (EUS)-guided ablative therapies as a means of locally remodeling the TME to enhance immune responsiveness. In particular, we focus on radiofrequency ablation (RFA), which induces tumor destruction and promotes systemic immune activation through antigen release and modulation of immunosuppressive pathways. These evolving strategies have the potential to expand therapeutic options for patients with PDAC by integrating locoregional ablation with systemic immunotherapy approaches.

## 1. Introduction

Pancreatic ductal adenocarcinoma (PDAC) is among the most aggressive malignancies, with a five-year survival rate of approximately 9% and a rising incidence in several countries [1,2]. In the United States, PDAC is currently the third leading cause of cancer-related death and is projected to become the second within the next decade [3]. Surgical resection remains the only definitive curative option for pancreatic cancer; however, fewer than 20% of patients are eligible at diagnosis due to metastatic disease [4] or locally advanced pancreatic cancer (LAPC), defined as non-metastatic disease with tumor involvement of major vessels precluding resection [5]. Even in operable cases, surgery is highly invasive and is associated with significant perioperative morbidity [6].

Chemotherapy, radiation, and surgery have been the mainstays of PDAC treatment for over two decades. However, despite their widespread use, these therapies have not significantly improved overall survival, largely due to late diagnosis, chemotherapy resistance, and high rates of recurrence [7]. In fact, up to 80% of patients will develop tumor recurrence within 5 years after PDAC resection [8]. Immunotherapy, while effective in other malignancies, has shown limited efficacy in PDAC. This is attributed largely to the tumor’s dense desmoplastic stroma, hypoxia, and profoundly immunosuppressive tumor microenvironment (TME). The TME comprises suppressive cell types such as regulatory T cells, tumor-associated macrophages, and myeloid-derived suppressor cells, all of which restrict immune cell infiltration, suppress effector functions, and blunt systemic treatment responses, resulting in poor response to immunotherapy [9]. These characteristics are why PDAC is often referred to as an immunologically “cold” tumor.

These unique characteristics of the TME pose formidable barriers to durable immune activation and have prompted growing interest in locoregional strategies that can directly disrupt the tumor niche. Thermal and non-thermal ablation techniques, including photodynamic therapy, cryoablation, irreversible electroporation (IRE), microwave ablation, and radiofrequency ablation (RFA) have been investigated as methods to induce tumor necrosis, expose antigens, and promote immune activation. Many of these modalities are being delivered under endoscopic ultrasound (EUS) guidance, offering a minimally invasive, anatomically precise approach that may be safer and more practical for patients who are not candidates for surgery [10,11,12,13]. Of note, ablation is generally considered in the setting of an unresectable tumor, metastatic disease, or for palliative purposes. Tumor resection is still preferred when possible.

Among these techniques, RFA has emerged as the most clinically accessible and widely studied approach for pancreatic tumors. EUS-guided RFA allows for precise, minimally invasive tumor ablation. Studies suggest that RFA can enhance immune infiltration and remodel the tumor microenvironment. Its favorable safety profile and increasing clinical data support its potential integration with immuno-oncologic strategies in PDAC [14,15].

This review aims to provide a comprehensive overview of endoscopic ablative therapies for pancreatic cancer, focusing on their principles, techniques, and emerging clinical applications. A particular emphasis is placed on radiofrequency ablation, the most extensively studied modality, for its potential to remodel the tumor microenvironment, promote immune activation, and enhance therapeutic outcomes. We also examine the immune-modulating effects of RFA within the tumor microenvironment, highlighting strategies that may prevent recurrence and overcome resistance in pancreatic ductal adenocarcinoma.

## 2. Endoscopic Platforms Enabling Ablation Therapy in PDAC

The evolution of endoscopic technologies has reshaped the management of pancreatic cancer, expanding their role from diagnostic tools to platforms for targeted intervention. Endoscopic retrograde cholangiopancreatography (ERCP) remains an essential tool for managing biliary obstruction in pancreatic cancer, while endoscopic ultrasound (EUS) has become the cornerstone for tissue acquisition and emerging locoregional therapies [16].

EUS allows real-time imaging and direct access to deep pancreatic lesions through the gastrointestinal wall, enabling highly precise interventions. Improvements in echoendoscope design and needle technology have extended its use beyond diagnostic biopsy to include therapeutic applications [17]. EUS-guided fine-needle injection (FNI) has become a valuable method for delivering therapeutic agents directly into pancreatic tumors, offering a localized approach that may help minimize systemic toxicity [18,19].

These advancements laid the foundation for EUS-guided tumor ablation. The same platform that allows for diagnostic biopsy and FNI can now deliver ablative energy—such as radiofrequency ablation (RFA) and irreversible electroporation (IRE)—directly into the tumor [14]. As these techniques gain clinical traction, EUS continues to evolve from a diagnostic modality into a gateway for novel immunomodulatory therapies.

## 3. Endoscopic Pancreatic Ablation: Techniques and Clinical Implementation

A variety of ablative therapies are used in the treatment of pancreatic cancer, each employing distinct approaches to local tumor destruction. These methods may also enhance immune activation and are increasingly being integrated into endoscopic oncology. The following sections highlight key modalities, outlining their mechanisms and clinical implementation (Table 1).

### 3.1. Photodynamic Therapy

Photodynamic therapy (PDT) is a non-thermal, localized ablative technique that combines systemic delivery of a tumor-selective photosensitizer with targeted light activation, typically at 630 nm. This generates cytotoxic species that induce localized tumor cell death via apoptosis and necrosis. Unlike thermal modalities, PDT preserves surrounding structures and minimizes inflammation. EUS-guided PDT enables precise fiber placement via a 19G fine needle aspiration (FNA) needle, allowing real-time tumor targeting in patients with unresectable LAPC [20]. One of the first clinical trials of PDT in pancreatic cancer was conducted by Bown et al. in 2002 using percutaneous light activation of mesotetrahydroxyphenylchlorin (mTHPC) in 16 patients with unresectable PDAC. Tumor necrosis was achieved in all patients, with a median survival of 9.5 months with a one-year survival rate of 44% and no treatment-related mortality [21]. Choi et al. reported the first clinical study of EUS-guided PDT in pancreatic cancer, enrolling four patients with locally advanced disease. The procedure was well-tolerated, with no treatment-related complications, and all patients demonstrated stable disease during a median follow-up of five months [22]. More recent studies have advanced EUS-PDT delivery. DeWitt et al. conducted a Phase I trial using EUS-guided porfimer sodium PDT in 12 patients. Tumor necrosis increased in 50% of cases, with median progression-free and overall survival of 2.6 and 11.5 months, respectively, with no serious adverse events reported [23]. A follow-up prospective study by Hanada et al. in 2021 using verteporfin-based PDT demonstrated the feasibility of repeated EUS-PDT sessions with favorable safety and tumor response rates [20]. These studies support PDT as a feasible endoscopic modality for localized tumor control in unresectable PDAC. Ongoing clinical trials will help define its role within combined therapeutic strategies.

### 3.2. Cryothermal Ablation

Cryoablation uses rapid freeze–thaw cycles to induce tumor cell death through direct mechanical injury and vascular disruption [24]. Cryoprobes cooled by argon or nitrogen gas form intracellular ice crystals, leading to membrane rupture, ischemia, and eventual cell necrosis [25]. Unlike thermal ablation, cryothermal injury can minimize collateral damage, particularly near sensitive structures including major vessels of the pancreatic duct. These features make it an appealing option for local control in patients with unresectable or medically inoperable pancreatic cancer [26]. Despite its appeal, cryoablation carries a notable risk of complications. In a prospective study of 22 patients undergoing EUS-guided cryothermal ablation, treatment was successfully delivered in 16 cases. Minor adverse events occurred in approximately 44% of treated patients, including transient amylase elevation, abdominal discomfort, and one case of duodenal bleeding that was managed endoscopically. Additionally, three patients (18.75%) experienced complications related to tumor progression. The median survival following treatment was six months [10]. Outcomes have been variable, and evidence remains limited to small studies and early-phase trials. Its clinical role continues to evolve, with ongoing efforts to refine technique, improve delivery, and explore combination strategies with systemic therapies.

### 3.3. Irreversible Electroporation

Irreversible electroporation (IRE) is a non-thermal ablation modality that delivers high-voltage, pulsed electric fields to tumor tissue, disrupting the integrity of cell membranes through permanent nanopore formation [27]. This leads to apoptosis and cell death without relying on heat, preserving the structural integrity of adjacent blood vessels, bile ducts, and connective tissue [28]. Since its initial introduction to oncology by Davalos et al. in 2005 [29], IRE has gained clinical traction as an adjunctive therapy for tumors located in anatomically challenging regions. In pancreatic cancer—particularly locally advanced pancreatic cancer (LAPC)—IRE offers a non-surgical method of achieving local tumor control in cases where curative resection is precluded by vascular involvement. Its ability to ablate tumor tissue without thermal damage makes it especially valuable in this context, and studies suggest it may enhance survival when integrated into multimodal treatment strategies [30]. In one of the largest prospective series to date, Martin et al. (2015) reported outcomes from 200 patients treated with IRE after neoadjuvant therapy [31]. The study demonstrated a median overall survival (OS) of 24.9 months from diagnosis and 23.2 months from the time of IRE, with a low 90-day mortality rate of 2%. Local progression-free survival reached 12.4 months, underscoring IRE’s capacity for durable regional control in otherwise unresectable disease [31].

Similarly, He et al. (2020) showed that patients treated with chemotherapy followed by IRE experienced significantly better outcomes than those treated with chemotherapy alone [32]. These findings support the use of IRE as a consolidative approach following systemic therapy, particularly in patients with stable or responding disease.

Interestingly, the CROSSFIRE trial (2024), which compared stereotactic ablative body radiotherapy (SABR) and CT-guided percutaneous irreversible electroporation, found no difference in overall survival between the two therapies [33]. Given these results, further research should be conducted before conclusions can be made on the effect of IRE on overall survival in PDAC.

Although IRE offers distinct advantages, its clinical application carries specific limitations. The delivery of long-duration electrical pulses (1–100 ms) can cause significant muscle contractions and carries a risk of cardiac arrhythmias. As a result, IRE requires general anesthesia, neuromuscular blockade, and electrocardiogram (ECG) synchronization to time pulse delivery with the cardiac refractory period [34]. While IRE holds promise as a complement to systemic therapy, its role remains under evaluation. Ongoing prospective studies will be critical to determining its optimal clinical application and long-term impact on survival.

### 3.4. Microwave Ablation

Microwave ablation (MWA) is a thermal ablative technique that utilizes electromagnetic waves, typically in the 900–2450 MHz frequency range, to induce rapid oscillation of water molecules within tissues. This process generates frictional heat, leading to coagulative necrosis of tumor cells [35,36]. Compared to other thermoablative modalities such as radiofrequency ablation (RFA), MWA can achieve higher intratumoral temperatures, larger and more uniform ablation volumes, and shorter procedural times due to reduced sensitivity to tissue impedance [37,38]. Originally applied in pulmonary and hepatic tumors in the early 2000 s, MWA has since expanded into gastrointestinal oncology, including pancreatic neoplasms [39]. In some studies, MWA has demonstrated feasibility and safety across various access routes, including open, laparoscopic, and percutaneous approaches. In a prospective study by Lygidakis et al., 15 patients with LAPC underwent open surgical MWA, achieving a 100% rate of partial tumor necrosis without major procedural complications [40]. Other studies reported high technical success rates and acceptable complication profiles with percutaneous MWA [39,41]. However, it is important to note these studies consist of small sample sizes. Given its thermal efficiency and adaptability across procedural platforms, MWA holds potential as a local therapy for pancreatic malignancy. However, validation in larger prospective trials is needed to define its efficacy, safety, and optimal role in endoscopic and non-surgical treatment pathways.

### 3.5. Radiofrequency Ablation

Radiofrequency ablation (RFA) is a minimally invasive, image-guided thermal ablation technique that uses a high-frequency alternating current, typically 350–500 kHz, to produce localized frictional heat, resulting in coagulative necrosis of neoplastic tissue while sparing adjacent normal structures [42,43]. RFA is one of the most extensively studied local ablative techniques in solid tumors and has been increasingly applied to PDAC, particularly in patients with locally advanced, unresectable disease where curative resection is not feasible.

RFA delivers a high-frequency alternating current through a targeted electrode to induce localized thermal injury. The standard system consists of a radiofrequency generator, an electrode needle, and either a monopolar or bipolar circuit [44]. In monopolar configurations, the current travels from the generator to a needle probe inserted into the tumor, then disperses through a grounding pad placed on the patient’s skin. The highest current density—and thus the greatest heat—is generated at the probe tip, producing temperatures of 60–100 °C and causing coagulative necrosis of tumor cells [45,46]. In bipolar systems, current flows between two closely spaced electrodes, eliminating the need for a ground pad and confining energy delivery to a localized area. This design enhances precision and limits collateral damage, making it particularly advantageous in the pancreas, where tumors often lie near critical vasculature and ducts [43,47].

EUS-guided RFA (EUS-RFA) has emerged as a preferred delivery platform in the pancreas due to the organ’s deep retroperitoneal location and proximity to critical vasculature. EUS-RFA allows for real-time visualization and precise targeting, reducing the risk of collateral injury. Various needle-based probes, typically 19G insulated electrodes with exposed tips, have been adapted for this purpose. Some feature internal cooling mechanisms to prevent charring and maintain consistent thermal delivery. These probes are typically classified into “through-the-needle” or “needle-type” configurations, with the latter offering better tip control and stability during ablation [42,48,49].

The earliest studies of pancreatic RFA were performed in porcine models by Goldberg et al., who confirmed that lesion sizes > 5 mm could be accurately ablated and visualized radiologically. Minor complications, such as gastric wall burns and elevated lipase, were observed but did not impact short-term survival in animals [50]. More recent preclinical studies, including orthotopic murine models of PDAC, have shown that RFA induces both local necrosis and immune cell infiltration—particularly dendritic cells and CD8+ T cells—suggesting potential for immune priming [42].

Clinically, RFA has demonstrated encouraging results in PDAC. A prospective study by Oh et al. evaluated 22 patients with unresectable pancreatic cancer who received EUS-RFA followed by gemcitabine-based chemotherapy. The authors reported a median overall survival of 24.03 months and a progression-free survival of 16.37 months, outcomes that surpass many chemotherapy-only regimens [51].

Across multiple series of studies, post-RFA survival has ranged from 13 to 25 months, depending on tumor size, treatment timing, and systemic therapy use [52,53]. An additional study of 26 patients found a significant improvement in performance status as well as significant tumor reduction [54].

Interestingly, Tieranu et al. conducted a review and meta-analysis which, after analyzing eleven studies, showed that there was a lack of consistent long-term survival between the studies [55]. Additionally, the PELICAN trial, which compared overall survival between groups receiving chemotherapy alone and RFA with chemotherapy, showed that there was no increase in survival in patients with locally advanced pancreatic cancer after receiving RFA in addition to chemotherapy [56]. Given the relatively small samples in these studies, larger studies should be performed to confirm whether there is any mortality benefit to RFA for these patients.

Regarding safety, EUS-RFA has shown a favorable profile. Reported complications include mild pancreatitis, peripancreatic fluid collections, duodenal wall injury, and, rarely, portal vein thrombosis or vascular pseudoaneurysms. Most complications are transient and resolve with conservative management [57]. Ongoing refinement of probe design and energy delivery parameters continues to enhance procedural safety.

In summary, RFA is currently the most clinically validated ablative modality in pancreatic cancer, with evidence supporting its safety, local efficacy, and potential to extend survival when used in conjunction with chemotherapy. Moreover, its immunostimulatory properties have prompted further investigation into its role as a synergistic partner to systemic immunotherapies—an emerging frontier explored in the following section.

Of note, while our review focuses on the treatment of PDAC, it is important to recognize that RFA has also been shown to be effective in the treatment of other pancreatic tumors such as neuroendocrine tumors (NETs). A review of sixty-one patients with pancreatic NETs found RFA to be 96% effective 11 months after treatment [58]. This review found no differences between functional and non-functional NETs. Barthet et al. reported similar results with 86% having tumor resolution at one year follow-up [59]. While the mainstay treatment for pancreatic NETs is surgery, the potential for complications such as pancreatic fistulas, delayed gastric emptying, and hemorrhage raises the need for alternative, minimally invasive therapies such as RFA.

**Table 1:** Comparison of pancreatic ablation techniques [20,21,22,23,24,25,26,27,28,29,30,31,32,33,34,35,36,37,38,39,40,41,42,43,44,45,46,47,48,49,50,51,52,53,54,55,56,57,58,59].

Modality	Mechanism	Delivery Method	Advantages	Limitations
Photodynamic Therapy (PDT)	Light-activated photosensitizer generates cytotoxic species causing apoptosis and necrosis	EUS-guided (via 19G FNA needle) or percutaneous approach	Minimally invasive, allows for localized and targeted therapy	Limited light penetration
Cryothermal Ablation	Freeze–thaw cycles create ice crystals and vascular injury causing cell death	EUS-guided cryoprobe	Less collateral structure damage, safer when operating near vessels	Potential for minor adverse events, evidence remains limited to small studies and early-phase trials
Irreversible Electroporation (IRE)	High-voltage electric fields create nanopores disrupting cell membranes, leading to apoptosis	Percutaneous approach, or open approach with ECG synchronization. Electrodes are placed around target tissue to deliver therapy	Non-thermal (minimizes damage to surrounding structures), safer near vessels	Requires general anesthesia and ECG sync, potential arrhythmias
Microwave Ablation (MWA)	Microwave-induced water molecule oscillation produces heat and coagulative necrosis	Open approach, laparoscopic, or percutaneous approach. Heat is delivered through ablation probe	Higher intratumoral temperatures, larger and more uniform ablation zones	Limited large-scale data, variable outcomes
Radiofrequency Ablation (RFA)	Alternating current generates heat causing coagulative necrosis via frictional heating	EUS-guided insulated needle electrodes	Minimally invasive, potential for immune activation and increased effectiveness with adjunctive chemotherapy	Complication risks (mild pancreatitis, vessel injury), requires precise targeting

## 4. Radiofrequency Ablation Effects on the Local Tumor Microenvironment

### 4.1. RFA as an Immune Primer in Pancreatic Cancer

In addition to local tumor destruction, RFA may exert immunomodulatory effects. The coagulative necrosis of tumor cells results in the release of tumor-associated antigens and signals, potentially enhancing systemic immune recognition. This property is of particular interest in PDAC, a tumor type typically resistant to immune checkpoint inhibition. The potential of RFA to convert immunologically “cold” tumors into more responsive phenotypes is a subject of ongoing research [60,61,62].

### 4.2. DAMP Release and Activation of Innate Immunity

The thermal injury produced by RFA (typically 60–100 °C) results in rapid tumor cell necrosis and the release of damage-associated molecular patterns (DAMPs), including ATP, calreticulin, and heat shock proteins (HSPs) [63]. These molecular signals activate dendritic cells (DCs) and promote antigen uptake and cross-presentation, leading to upregulation of MHC class I molecules and enhanced tumor antigen visibility to CD8+ T cells. Concurrently, RFA induces the release of pro-inflammatory cytokines such as IL-6, TNF-α, and CXCL1, further amplifying the innate immune response [64].

### 4.3. Neutrophil Infiltration and the Innate–Adaptive Bridge

A notable feature of RFA-induced immune modulation is the early infiltration of neutrophils into the ablated zone [64]. These neutrophils, particularly those exhibiting an N1-like (anti-tumor) phenotype, secrete reactive oxygen species (ROS), TNF-α, and chemokines that attract both DCs and cytotoxic T cells [65]. This innate–adaptive immune bridging appears crucial for generating sustained anti-tumor activity and may partially overcome the immunologically “cold” landscape of PDAC.

### 4.4. Neutrophil-Driven Immune Activation

Neutrophils play a central role in shaping the early immune response following tumor ablation. In the setting of RFA, studies have shown a marked increase in neutrophil infiltration into the TME, contributing to both local inflammation and structural remodeling [65]. Beyond promoting neutrophil accumulation, RFA has also been associated with reductions in immunosuppressive populations, including regulatory T cells (Tregs) and tumor-associated macrophages (TAMs), shifting the TME toward a more pro-inflammatory, anti-tumor phenotype [66]. Timing appears to be critical in neutrophil-mediated immune activation. Preclinical models demonstrated that tumor-associated neutrophils (TANs) peaked approximately 24 h after ablation therapy [67]. Depletion of neutrophils during this window resulted in accelerated tumor growth, emphasizing their essential role in controlling tumor progression post treatment. Cytokines such as G-CS and IL-1β have been implicated in neutrophil activation, with G-CSF levels notably peaking at 24 h after therapy [67]. Further supporting these findings, Faraoni et al. demonstrated that RFA-induced neutrophil infiltration correlated with systemic immune activation, including enhanced recruitment of cytotoxic T lymphocytes (CTLs) and natural killer (NK) cells, consistent with the development of abscopal-like responses [65].

### 4.5. The Abscopal Effect: Local Ablation, Systemic Response

**Figure 1:**
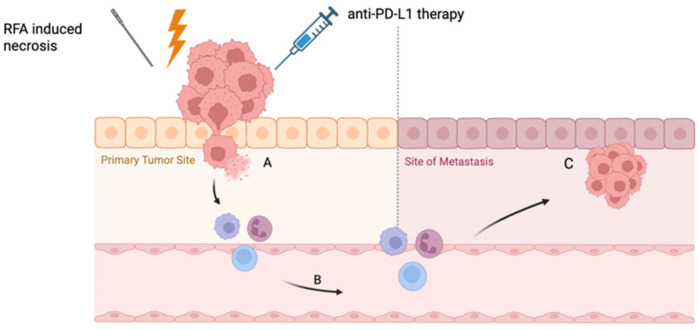
The abscopal effect: localized therapy leading to regression of distant metastasis. A: RFA induced tumor necrosis and antigen release; B: enhanced immune system activation and migration of immune cells to distant metastases after RFA + anti-PD-L1 treatment; C: decreased growth of metastatic cancer [42,53,66,68].

The resulting necrosis from thermal ablation not only disrupts tumor architecture but also releases cellular debris and tumor-associated antigens, serving as a potent source of immune stimulation that may propagate systemic anti-tumor responses. This phenomenon—where localized therapy leads to regression of distant, untreated lesions—is known as the abscopal effect (Figure 1) [42].

Evidence of abscopal-like responses following radiofrequency ablation (RFA) is emerging in both preclinical and clinical settings. Thosani et al. described a patient with metastatic ampullary carcinoma who survived 73 months post diagnosis following RFA, far exceeding expected outcomes, and hypothesized that systemic immune activation contributed to this prolonged survival [53]. Similarly, murine models have demonstrated that ablation of a primary pancreatic tumor can enhance immune cell infiltration at distant metastatic sites, supporting the concept that local tumor destruction can initiate broader immune activation [68].

The abscopal effect is thought to be mediated by the release of tumor antigens and DAMPs from necrotic cells, leading to dendritic cell maturation, cross-presentation of tumor antigens, and activation of cytotoxic T lymphocytes (CTLs). Additionally, ablation-induced inflammation can enhance trafficking of effector T cells to both local and distant tumor sites. Given the traditionally immunologically “cold” nature of pancreatic tumors, RFA’s ability to trigger systemic responses provides a compelling strategy to enhance the efficacy of immune checkpoint blockade and other systemic therapies [66]. Although clinical demonstrations of the abscopal effect in PDAC remain limited, growing preclinical data underscore the potential of RFA not only as a local therapy but also as a catalyst for systemic immune engagement.

### 4.6. RFA-Induced PD-L1 Expression: A Rationale for Immune Checkpoint Inhibition

PD-L1 is a receptor found on various cells, but usually in higher quantities on cancer cells, that binds with PD-1 receptors on T cells to ultimately dampen immune responses. Interestingly, PDAC typically exhibits low baseline PD-L1 expression and therefore has limited responsiveness to PD-L1 inhibitors. However, RFA has the potential to alter this paradigm. The inflammatory response following ablation can induce interferon-γ signaling, which results in the upregulation of PD-L1 on tumor and stromal cells. Several preclinical studies have demonstrated that this post-ablation increase in PD-L1 expression sensitizes tumors to immune checkpoint inhibitors (ICIs), particularly those that target PD-L1 [42]. Immune checkpoint inhibitors are monoclonal antibodies that inhibit the regulatory pathways of T cell activation by targeting receptors such as PD-1 and PD-L1. In doing so, they allow for unchecked T cell proliferation and an enhanced immune response against tumor cells [69]. While ICIs are usually not significantly effective for treatment of PDAC, administering ICIs—specifically anti–PD-1 or anti–PD-L1 therapies—during the post-RFA inflammatory window may block adaptive immune suppression and sustain cytotoxic T cell activity, improving outcomes in a classically immune-resistant tumor [66,70,71]. Of note, studies on radiation therapy for local tumors have also found that there is increased tumor suppression when ICI therapy is administered within five days for radiation [72]. While it has not yet been studied, there is likely a specific window of time in which the activation of dendritic cells and inflammatory cytokines results in the upregulation of PD-L1 after RFA before baseline PD-L1 expression returns. Further studies are needed to confirm and determine the ideal time frame in which ICI therapy should be administered after RFA for optimal results.

### 4.7. Adenosine and CD73: A Targetable Immunosuppressive Escape

Although RFA promotes immune activation, compensatory immunosuppressive mechanisms, particularly the adenosine (ADO) pathway, may limit the durability of the antitumor response. In this pathway, increased extracellular ATP released during DAMP signaling is rapidly converted to immunosuppressive adenosine via the CD39/CD73 enzymatic cascade. Adenosine acts on A2A receptors to inhibit effector T cell function and dampen anti-tumor immunity [73]. Upregulation of the ADO pathway following RFA has been implicated in T cell dysfunction and tumor progression. As a result, targeting the adenosine axis—particularly with anti-CD73 antibodies—has emerged as a promising strategy to enhance the efficacy of RFA and sustain immune activation. Preclinical studies demonstrate that CD73 blockade enhances T cell function and works synergistically with RFA to prevent tumor recurrence, supporting the development of combined-based immune therapies [74].

## 5. Conclusions

By remodeling the tumor microenvironment and enhancing antigen exposure, radiofrequency ablation (RFA) is increasingly becoming recognized as an in situ tumor vaccine capable of converting immune-silent pancreatic tumors into immunologically active targets [75]. Coupling RFA with immune checkpoint inhibitors and adenosine-CD73 blockade offers a promising strategy to overcome the intrinsic immune resistance of pancreatic ductal adenocarcinoma (PDAC). In addition, EUS-guided fine-needle injection (FNI) enables precise intratumoral delivery of dendritic cells, cytokines, and oncolytic viruses, further enhancing local immune priming [14].

The growing body of preclinical and clinical evidence supporting RFA as an immune-potentiating modality has laid the foundation for integrated therapeutic strategies aimed at combining locoregional and systemic immune activation. Moving forward, future efforts and trials should focus on defining optimal markers of response to this novel treatment. In addition, there should be an emphasis on determining optimal delivery, timing, and dosing of ICIs, adenosine pathway inhibitors, and intratumoral therapies. As evidence in this field grows, advances in ablation platform technology and immunologic motioning should follow.

Endoscopic RFA represents a paradigm shift in the local and systemic management of pancreatic cancer. Beyond its cytoreductive capabilities, RFA promotes antigen release, innate and adaptive immune activation, and improved tumor visibility to systemic therapy. As the most clinically validated and technically feasible ablative strategy in this setting, RFA is positioned to become a foundational platform for advancing endoscopic immuno-oncology.
